# NPM/ALK mutants resistant to ASP3026 display variable sensitivity to alternative ALK inhibitors but succumb to the novel compound PF-06463922

**DOI:** 10.18632/oncotarget.3122

**Published:** 2015-01-30

**Authors:** Luca Mologni, Monica Ceccon, Alessandra Pirola, Gianpaolo Chiriano, Rocco Piazza, Leonardo Scapozza, Carlo Gambacorti-Passerini

**Affiliations:** ^1^ University of Milano-Bicocca, Dept. of Health Sciences, Monza, Italy; ^2^ University of Geneva, School of Pharmaceutical Sciences, Geneva, Switzerland; ^3^ San Gerardo Hospital, Hematology Unit, Monza, Italy

**Keywords:** NPM/ALK, inhibitor, resistance, ASP3026, PF-06463922

## Abstract

ALK is involved in the onset of several tumors. Crizotinib (Xalkori^TM^), a potent ALK inhibitor, represents the current front-line treatment for ALK+ NSCLC and shows great clinical efficacy. However, resistant disease often develops after initial response. ASP3026 is a novel second-generation ALK inhibitor with activity on crizotinib-resistant ALK-L1196M gatekeeper mutant. As resistance is likely to be a relevant hurdle for any drug, we sought to determine the resistance profile of ASP3026 in the context of NPM/ALK+ ALCL. We selected six ASP3026-resistant cell lines by culturing human ALCL cells in the presence of increasing concentrations of drug. The established resistant cell lines carry several point mutations in the ALK kinase domain (G1128S, C1156F, I1171N/T, F1174I, N1178H, E1210K and C1156F/D1203N were the most frequent) that are shown to confer resistance to ASP3026 in the Ba/F3 cell model. All mutants were profiled for cross-resistance against a panel of clinically relevant inhibitors including ceritinib, alectinib, crizotinib, AP26113 and PF-06463922. Finally, a genetically heterogeneous ASP3026-resistant cell line was exposed to second-line treatment simulations with all inhibitors. The population evolved according to relative sensitivity of its mutant subclones to the various drugs. Compound PF-06463922 did not allow the outgrowth of any resistant clone, at non-toxic doses.

## INTRODUCTION

Cancers are heterogeneous masses that evolve following population dynamics [1]: descendants from the founder cell expand according to their genotype and to the environment, which provides selective pressure. Drugs used to treat a tumor are part of its environment and represent a strong selective agent. Notwithstanding enormous clinical success of rationally targeted therapies, the emergence of drug-resistant subclones is a direct consequence of cancer heterogeneity and represents a formidable challenge toward definitive cure. Examples of this phenomenon abound in the literature, from initial pioneering observations in imatinib-resistant BCR/ABL-positive leukemia [2] to more recent cases in both hematological and solid tumors [3, 4]. Anaplastic Lymphoma Kinase (ALK)-driven tumors are no exception. ALK-related diseases comprise a diverse set of malignancies, including subsets of non-small cell lung cancer (NSCLC), anaplastic large-cell lymphoma (ALCL), inflammatory myofibroblastic tumor (IMT), neuroblastoma, as well as breast, colon and thyroid cancers, and other rarer diseases [5]. Aberrant ALK kinase activation is turned on by either point mutation or gene fusion and inappropriate ALK-driven signaling is known to cause malignant transformation. Consequently, ALK-dependent tumors are extremely sensitive to ALK inhibition: indeed, the recent introduction of crizotinib has been a major breakthrough in the management of ALK-positive cancer [6]. However, following anti-ALK-specific therapy, relapses frequently occur. Crizotinib-resistant disease is often associated with ALK kinase mutations or amplification, that render the enzyme refractory or less sensitive to the drug [7–11]. Second generation ALK inhibitors (ALKi) have been developed in order to overcome resistance to crizotinib [5]. However, we can expect resistance to arise under any treatment. In order to predict the possible evolution of ALK+ ALCL under ALKi pressure, and to study cross-resistance among the available drugs, we recently set up an *in vitro* model of acquired resistance to selective ALK inhibition in ALCL [12, 13]. As part of this effort, we isolated six NPM/ALK+ cell lines that show high resistance to the novel ALKi, ASP3026 (Astellas Pharma, Japan). ASP3026 was disclosed for the first time in 2011 [14, 15]. Recently, additional preclinical and clinical data were released [16–18]. The compound showed improved ALK selectivity compared to crizotinib and was able to suppress EML4/ALK-L1196M mutant xenografts *in vivo*, with no apparent toxicity. In a phase 1 trial, ASP3026 achieved 44% partial responses and 50% stable disease in patients who had progressed on crizotinib [18].

In this work, we describe ASP3026-resistant ALCL cells carrying novel single as well as double ALK kinase domain mutations. We further characterized the effects of these mutations on ALKi sensitivity in a Ba/F3 cell model and studied the evolution of a composite pool of ASP3026-resistant cells under second-line treatment with clinically relevant ALKi drugs [6, 19–22].

## RESULTS

### Selection of ASP3026-resistant cell lines

**Figure 1:**
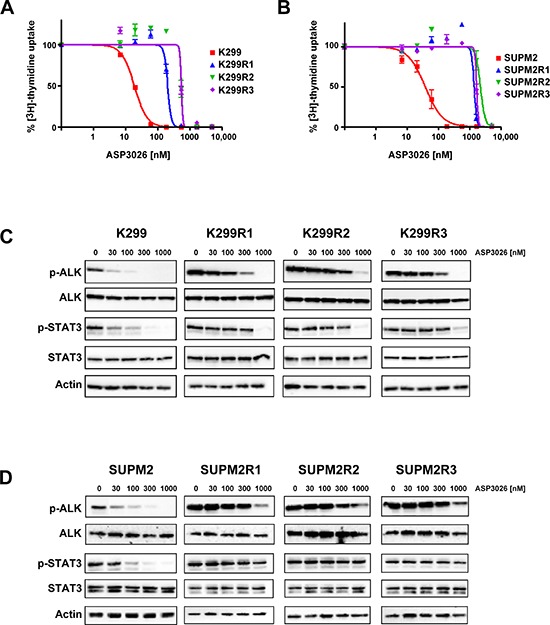
Characterization of ASP3026-resistant cells **(A–B)** Dose-response curves generated by ^3^H-Thymidine incorporation assay with native cells and drug-resistant cell lines. Karpas299 (K299) and Karpas299-derived resistant cells (K299R1, R2, R3) are shown in panel A. SUPM2 cells and their derived resistant clones are shown in B. The calculated IC_50_ values are reported in Table 1. **(C–D)** Western blot analysis of ALK and STAT3 phosphorylation inhibition by ASP3026, at the indicated nanomolar concentrations, in parental and resistant cells. Panel C, K299; panel D, SUPM2. Total ALK, STAT3 and actin are shown for loading normalization.

Two human NPM/ALK (N/A)-expressing ALCL cell lines were employed for the selection of ALKi-resistant clones: Karpas-299 (K299) and SUPM2. Three independent populations were derived from each parental line and cultured in the presence of ASP3026, starting from concentrations near the observed cell growth IC_90_ (for K299, 100 nM; for SUPM2, 200 nM; see Figure 1). Cell viability and growth rate dropped accordingly (Supplementary Figure S1). When the cell populations appeared to regain a normal growth rate, after approximately 2–3 weeks, the concentration of the drug was increased. After sequential stepwise increases (over a total period of two to three months), three K299 populations (K299R1, K299R2, K299R3) that grew at 0.5 μM ASP3026 (25-fold the IC_50_ of the original line) were obtained; similarly, three SUPM2 cell lines growing at 2 μM ASP3026 (50-fold the parental cells IC_50_) were selected (SUPM2R1, SUPM2R2, SUPM2R3). K299 cells could not survive ASP3026 concentrations higher than 0.5 μM. The established drug-resistant cell populations were characterized in terms of cell proliferation/viability compared to the original cell lines. While, as expected, parental cells growth was completely suppressed by ASP3026 over a time-course of 5 days, resistant cells were not only unaffected but they grew even better in presence of the inhibitor (Supplementary Figure S2A). Interestingly, soft-agar colony assays suggested that resistant cell lines had on average a decreased anchorage-independent growth potential compared to parental cells (Supplementary Figure S2B). However, the number of colonies was either unchanged or even increased by the presence of ASP3026 in the medium. In the case of SUPM2R1, colonies were observed only in the presence of the drug, suggesting a sort of drug-addiction. Sensitivity of the selected cells to ASP3026 was then analyzed by cell proliferation using dose-response curves. As shown in Figure 1A–1B and in Table 1, the six resistant cell lines showed a 10- to 60-fold shift in IC_50_, compared to their parental counterparts. To verify that decreased sensitivity to the inhibitor was indeed due to persistent N/A kinase activity, the cells were challenged with increasing doses of ASP3026, and N/A tyrosine phosphorylation (pALK) was measured, as an indicator of enzyme activation (Figure 1C–1D). While pALK signal was dramatically reduced by 30–100 nM in parental cells, all resistant cell lines showed persistent ALK phosphorylation at 300 nM and in some cases up to 1000 nM. A parallel change in STAT3 phosphorylation pattern indicates that downstream signaling is affected in a similar manner, in resistant cells. These observations suggest that the selected populations are able to maintain an active N/A oncogenic signal in the presence of ASP3026 concentrations that normally cause complete suppression of K299 and SUPM2 cells growth, and this translates into the ability to proliferate normally. Interestingly, both SUPM2 and K299 resistant cells showed an increased basal pALK band compared to parental cells. This may be achieved either by N/A overexpression or by increased intrinsic activity of mutant N/A. As shown in Figure 1, K299R cells showed a slight increase of total ALK band intensity. Using a different anti-ALK antibody, we confirmed that K299R cells express approximately 5 to 9-fold more ALK protein, as determined by densitometry analysis, while SUPM2R cells showed only a modest (2–4x) increase (Supplementary Figure S3). Real-time quantitative PCR confirmed the data in SUPM2R cells, but not in K299R cells, where N/A mRNA was only slightly increased (2 to 4-fold) in two out of three cell lines (Table 1). For a comparison, in other ALKi-resistant K299 cells carrying wild-type ALK sequence, we observed 16 to 25-fold increases in N/A mRNA expression and this was a clear effect of oncogene amplification [13]. Therefore, in this case, we cannot definitely ascribe resistance to oncogene overexpression.

**Table 1:** Characterization of ASP3026-resistant cell lines **Cell proliferation data, qPCR, sequencing analyses and mutant frequency within each population are shown**. Frequency is calculated from chromatogram peak height, except in SUPM2R3, where the actual prevalence of TOPO-TA clones is reported (23 clones sequenced; see Methods section).

**Cell line**	**ASP3026 IC50 [nM]**	**IC50 fold increase (RR)**	**NPM/ALK transcript level (normalized)**	**Mutation (NM_004304.4)**	**Aminoacid substitution**	**Frequency (%)**
**K299**	19	1	1	-	-	
**K299R1**	208	11	3.8	4484A > C	N1178H	100
**K299R2**	552	29	2.0	4463T > C	I1171T	~50
**K299R3**	550	29	0.8	4484A > C 4418G > A	N1178H C1156Y	~40 ~15
**SUPM2**	38	1	1	-	-	
**SUPM2R1**	1381	36	2.7	4463T > A	I1171N	~70
**SUPM2R2**	2186	58	2.4	4334/4335 GG > TC	G1128S	100
**SUPM2R3**	1654	44	2.5	4418G > T/4559G > A 4580G > A 4472T > A 4418G > T other*	C1156F/D1203N E1210K F1174I C1156F other*	43 39 9 4 4

* *other*, refers to a complex deletion within the kinase domain, possibly an artifact of cloning, observed in 1/23 clones.

### Identification of NPM/ALK mutations associated with resistance

Next, the entire kinase domain of N/A was sequenced in K299, SUPM2 and their respective ASP3026-resistant subpopulations, by standard Sanger method. As reported in Table 1 and Supplementary Figure S4, several mutations were identified that may explain the observed biological resistance to the treatment. Interestingly, K299R2 and SUPM2R1 carry different changes at the same position (I1171T and I1171N, respectively), while SUPM2R2 cells harbor a double nucleotide substitution within the same codon, leading to G1128S aminoacid change. The SUPM2R3 population is clearly a pool of different subclones, as we observed three mutations at approximately 50% relative peak intensity. K299R3 did not appear to carry ALK mutations at detectable frequency, by standard sequencing. However, by ultradeep sequencing, several mutations were identified (Supplementary Table 1). In particular, N1178H and C1156Y substitutions were present in 49% and 23% of the clones, respectively, and were later validated by Sanger method (Supplementary Figure S4). The well-known gatekeeper L1196M mutant was also detected at low frequency (6.8%) by deep sequencing. In order to verify that the double mutation in SUPM2R2 indeed occurred *in cis*, and to calculate the relative frequency of SUPM2R3 mutants, clonal sequencing was carried out after subcloning of N/A amplicons. SUPM2R2 double mutation was confirmed to be on the same filament, in 25/25 (100%) clones, while SUPM2R3 cells proved to be a mixture of at least 4 different mutants, including a double mutation (C1156F/D1203N) that was present in the same filament in 10/23 clones (Table 1). SUPM2R3 were further analyzed by deep sequencing, confirming these data (Supplementary table 2). Interestingly, a small fraction of SUPM2R3 clones (4.9%) carried the I1171S aminoacid change. Thus, in our set of ASP3026-resistant cells, at least three different I1171 substitutions were selected.

### Characterization of NPM/ALK mutants sensitivity to ASP3026 in the Ba/F3 system

**Figure 2:**
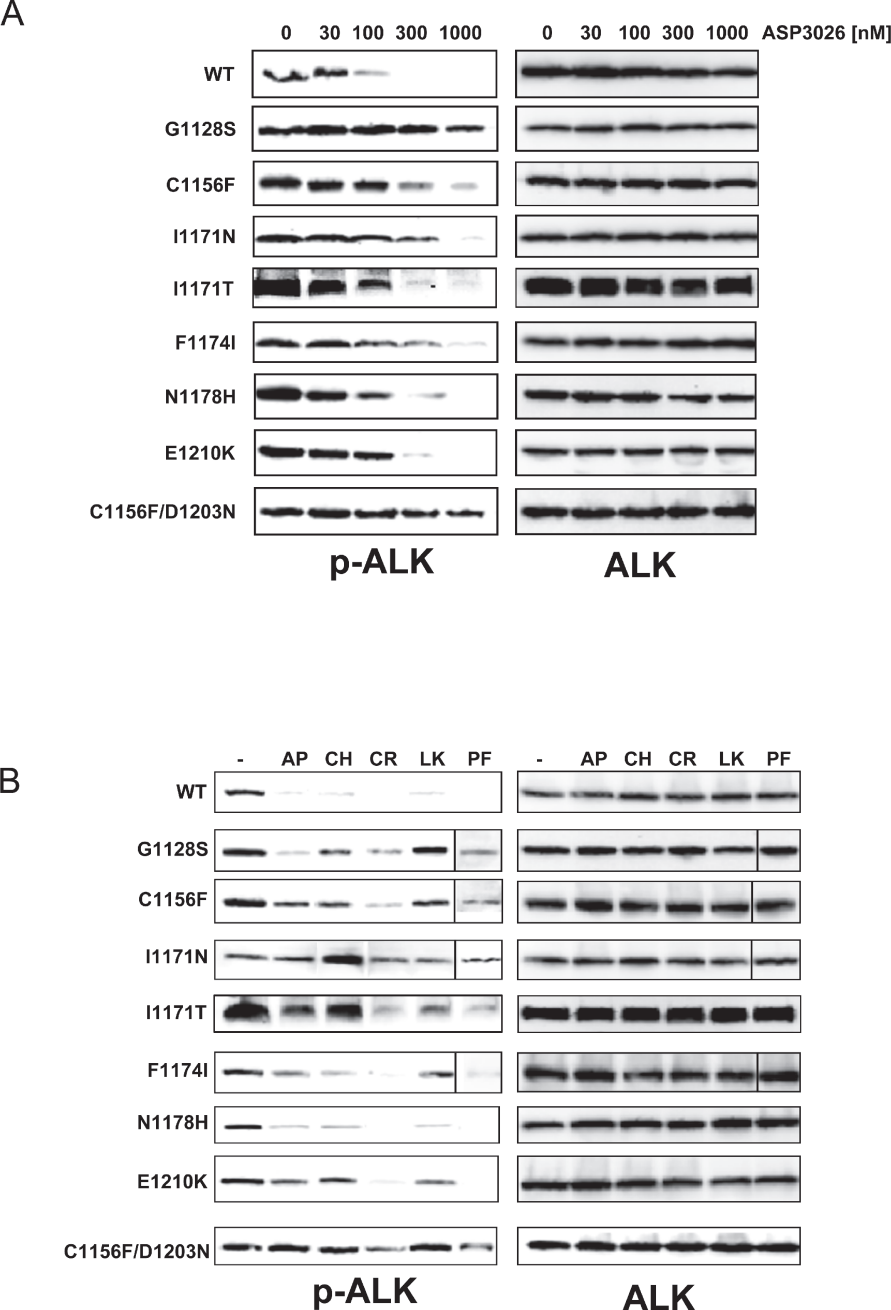
Analysis of Ba/F3-NPM/ALK cell lines sensitivity to ALK inhibitors **(A)** Western blot analysis of ALK phosphorylation inhibition by ASP3026, at the indicated nanomolar concentrations, in wild-type and mutant Ba/F3-NPM/ALK cell lines. **(B)** Analysis of phospho-ALK inhibition by low-dose ALK inhibitors. The lowest inhibitors concentrations causing complete suppression of pALK signal in WT cells were used. AP, 10 nM AP26113; CH, 10 nM alectinib; CR, 300 nM crizotinib; LK, 30 nM ceritinib; PF, 10 nM PF-06463922. Total ALK is shown on the right-hand side of panels A and B, as loading control.

The most frequent mutations identified in ASP3026-resistant cell lines were re-introduced in the wild-type (WT) N/A sequence by site-directed mutagenesis and expressed in Ba/F3 cells, an IL-3-dependent murine cell line that acquires interleukin independence upon oncogene expression [12]. The C1156Y and L1196M mutants have already been extensively investigated [7, 10, 12, 23, 24] and were not analyzed further. All transfected cells (hereafter referred to as BaF3-N/A) expressed the transgene (Supplementary Figure S5A–S5B). Expression of the correct mutation was verified by sequencing the N/A transcript (Supplementary Figure S6). We then evaluated the sensitivity of BaF3-N/A mutants to ASP3026, compared to WT and to parental IL-3-dependent Ba/F3, both in cell proliferation assays (Tables 2–4) and by pALK Western blot (Figure 2A). To summarize cell growth data, the IC_50_ value obtained for each cell line is reported in Table 2. Moreover, we calculated for each mutant a relative resistance (RR) index, as the IC_50_ fold increase compared to cells carrying WT N/A (Table 3). This parameter gives an estimate of the impact that a mutation has on enzyme sensitivity to a drug [25]. The higher the number, the more a particular mutant is resistant to treatment, relative to wild-type. All mutants showed an increase of ASP3026 IC_50_ compared to WT (RR = 2.4–40), thus supporting the hypothesis that the identified mutations are able to confer resistance to ASP3026 (Tables 2–3). These results were confirmed by ALK phosphorylation data (Figure 2A). In general, there was some variability among the various mutants. In particular, the double mutant C1156F/D1203N showed the highest resistance index in both assays, while N1178H was the least resistant, combining results from the two tests. We could not establish an IL3-independent line expressing the single D1203N mutation, in two separate attempts. This may be due to low intrinsic activity of this N/A mutant.

**Table 2:** IC_50_ values [nanomolar units] obtained with parental and N/A-transfected Ba/F3 cells treated with the indicated inhibitors **The NPM/ALK aminoacid substitutions are shown in the first column**. WT = wild-type NPM/ALK. The data (mean ± SD) represent the average of three or more independent experiments.

	**ASP3026**	**AP26113**	**alectinib**	**crizotinib**	**ceritinib**	**PF-06463922**
Parental (+IL3)	4252 ± 976	1210 ± 56	444 ± 14	2690 ± 180	1586 ± 173	2968 ± 39
WT	70 ± 6	6.7 ± 0.5	3.8 ± 0.6	41 ± 8	21 ± 8	1.2 ± 0.1
G1128S	1022 ± 155	4.9 ± 1	8.2 ± 1	140 ± 37	102 ± 38	12 ± 1
C1156F	1293 ± 360	143 ± 46	149 ± 40	80 ± 16	217 ± 115	183 ± 13
I1171N	519 ± 199	35 ± 2	108 ± 58	251 ± 89	187 ± 87	11 ± 9
I1171T	445 ± 17	51 ± 9	73 ± 8	95 ± 13	82 ± 12	173 ± 1
F1174I	184 ± 46	6.2 ± 2	3.2 ± 1	48 ± 3	13 ± 0.1	2.2 ± 0.1
N1178H	169 ± 76	17 ± 8	4.5 ± 0.2	47 ± 7	42 ± 6	2.9 ± 0.3
E1210K	748 ± 211	103 ± 42	120 ± 19	99 ± 31	187 ± 84	67 ± 30
C1156F/D1203N	2809 ± 414	67 ± 7	15 ± 1	570 ± 174	254 ± 99	64 ± 18

**Table 3:** Relative Resistance indexes **The IC_50_ fold increase obtained with mutant BaF-N/A cells is reported (wild-type N/A [WT] = 1), calculated from data in Table 2**.

	**ASP3026**	**AP26113**	**alectinib**	**crizotinib**	**ceritinib**	**PF-06463922**
Ba/F3 parental	41	179	116	59	77	2508
WT	1	1	1	1	1	1
G1128S	15	0.7	2.1	2.8	5.0	10
C1156F	19	21	39	1.6	11	155
I1171N	7.4	5.2	28	5.0	9.1	9.5
I1171T	6.4	7.5	19	2.3	3.99	146
F1174I	2.6	0.9	0.8	1.0	0.6	1.9
N1178H	2.4	2.5	1.2	0.9	2.1	2.4
E1210K	11	15	31	1.97	9.1	57
C1156F/D1203N	40	10	4.0	11	12	54

**RR legend**	
< 2	Sensitive
2–4	Slightly resistant
4–10	Fairly resistant
> 10	Highly resistant

**Table 4:** Therapeutic Indexes **The ratio between IC_50_ of Ba/F3 parental and mutant BaF-N/A cells is reported (Ba/F3 = 1), calculated from data in Table 2**. WT = wild-type NPM/ALK.

	**ASP3026**	**AP26113**	**alectinib**	**crizotinib**	**ceritinib**	**PF-06463922**
Ba/F3 parental	1	1	1	1	1	1
WT	41	179	116	59	77	2508
G1128S	2.8	245	54	21	15	245
C1156F	2.2	8.4	3.0	37	7.3	16
I1171N	5.5	35	4.1	12	8.5	265
I1171T	6.4	24	6.1	31	19	17
F1174I	15	194	139	61	121	1334
N1178H	17	72	99	62	37	1029
E1210K	3.8	12	3.7	30	8.5	44
C1156F/D1203N	1.0	18	29	5.2	6.2	46

**TI legend**	
> 20	Druggable
10–20	Fairly druggable
5–10	Poorly druggable
< 5	Undruggable

From a therapeutic standpoint, there are two options when we are confronted with drug resistance: either to increase the dosage, or to change regimen. Dose increase is achievable within the limits of unspecific toxicity. To get an approximate view of this window, we calculated the ratio between IC_50_ of parental Ba/F3 cells (which should represent unwanted off-target effects) and IC_50_ of BaF-N/A cells. We define this ratio as ‘therapeutic index’ (TI; Table 4), that is, how much more sensitive is a mutant compared to non-target cells, as reported by Sakamoto et al. [19]. In this case, the lower the index value, the more a mutant resembles parental ALK-independent Ba/F3 cells, indicating a poor therapeutic window. Two mutants, F1174I and N1178H, showed a large TI (> 10) suggesting that such mutants may be neutralized by a raise in ASP3026 dosage. In fact, these two mutants also showed the smallest RR index. All other mutants are indeed problematic, since their sensitivity to ASP3026 is too close to that of parental Ba/F3 cells (i.e., they have a low TI).

### Cross-resistance of NPM/ALK mutants against a panel of ALK inhibitors

When planning a change of drug, one would ideally want to know in advance the sensitivity of refractory disease to the proposed second-line regimen. In order to mimic such a situation, we tested the sensitivity of all mutants to currently available ALK inhibitors. The results are reported in Tables 2–4 (IC_50_, RR, TI values, respectively) and in Figure 2B (pALK data) and suggest that F1174I and N1178H are the most easily tractable mutants, as they show sensitivity to most second-generation inhibitors. BaF-N/A cells carrying the G1128S substitution displayed variable sensitivity to the various drugs and should not represent a big challenge in the future. Both C1156F and E1210K mutants were sensitive to crizotinib, while I1171N and the double C1156F/D1203N mutations showed resistance to all drugs, although at varying degrees (Table 3). Interestingly, crizotinib efficacy on several mutants was comparable to WT cells, indicating that it was not greatly affected by ASP3026-selected mutations: apparently, ASP3026 and crizotinib have little overlap in their resistance profiles. To confirm this finding, human cells that had been previously selected by crizotinib [12] were cross-tested with ASP3026. As expected, crizotinib-resistant cells carrying a L1196Q substitution were highly sensitive to ASP3026, while I1171N-mutated cells were resistant (Supplementary Figure S7A–S7C). To give a more translational view of these results, RR index data are complemented by TI values: despite high RR values, each mutant may be targeted by at least one drug at concentrations that are still far from unspecific toxicity (Table 4). For example, the double C1156F/D1203N was sensitive to nanomolar concentrations of alectinib and PF-06463922, far below the IC_50_ observed in Ba/F3 cells. In particular, the results obtained with PF-06463922 illustrate this point: because of the very large window of Ba/F3 versus BaF-N/A-WT sensitivity (> 2500x) some mutants that display high RR values (shift in sensitivity compared to WT) also show a high TI, suggesting that they may be treated with a tolerable compound dose. Otherwise, TI values confirmed that F1174I and N1178H are the weakest mutants, G1128S shows good druggability with other compounds, while all others are on average poorly druggable, although for each mutant there is at least one inhibitor that has a large TI (Table 4).

### Second-line treatments of ASP3026-resistant cells

**Figure 3:**
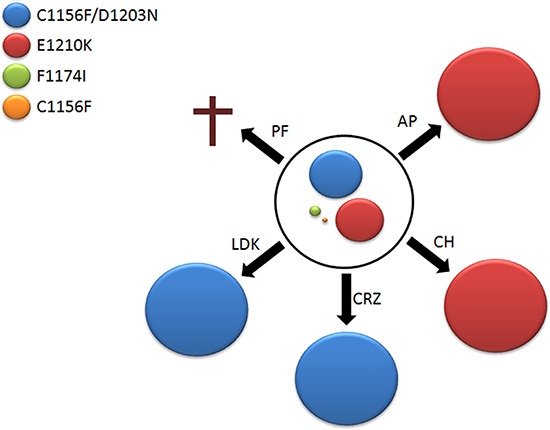
Second-line therapy ASP3026-resistant SUPM2R3 cells were exposed to equitoxic doses of ALK inhibitors. As secondary drug-resistant populations emerged, ALK sequence was determined. The circle in the center represents SUPM2R3 polyclonal population, with mutants shown as colored discs whose size is proportional to mutant frequency within SUPM2R3 pool. Black arrows indicate treatments: AP, 240 nM AP26113; CH, 90 nM alectinib; CRZ, 600 nM crizotinib; LDK, 320 nM ceritinib; PF, 300 nM PF-06463922. The selected mutants are shown according to color code indicated in the figure. A cross at PF-06463922 indicates that no clone grew under these conditions.

As tumors develop drug-resistant clones, they may face additional therapies and therefore evolve in different directions according to the selective pressure they undergo. We simulated second-line treatments of the SUPM2R3 heterogeneous population with all other drugs. The cells were treated for two weeks with concentrations corresponding to 20% of the Ba/F3 parental cell line IC_50_, which was taken as a reference for unspecific toxicity. Interestingly, cells exposed to alectinib and AP26113 evolved a homogeneous clone carrying the E1210K mutation in 100% of the cells (Figure 3 and Supplementary Figure S8), in line with proliferation data that indicate high resistance to these inhibitors by N/A-E1210K. On the other hand, a pure C1156F/D1203N double mutant emerged under crizotinib and ceritinib, in agreement with data showing that crizotinib can easily suppress E1210K but not the double mutation. Finally, when challenged with the corresponding dose of PF-06463922, no resistant cells could be retrieved. When the selection was repeated at half dose, again no outgrowth was observed. Thus, PF-06463922 was the only inhibitor able to efficiently overcome ASP3026 resistance in a highly heterogeneous cell population, at tolerable concentrations.

### Molecular modelling analysis of NPM/ALK mutations

**Figure 4:**
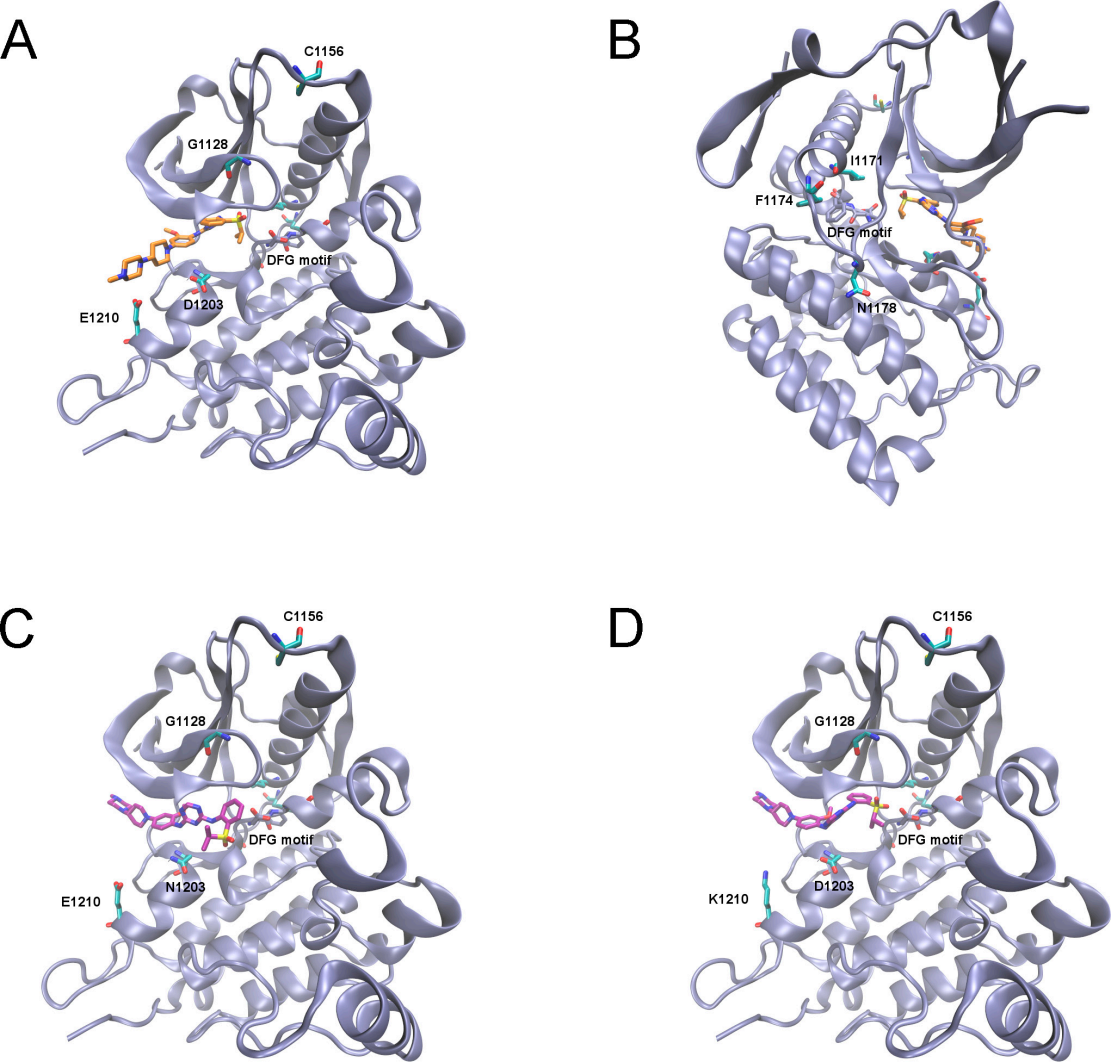
Low energy docking model of ASP3026 within the active site of ALK (PDB code: 4MKC) **(A–B)** Rotated views of ASP3026 (C atoms shown in orange) docking into ALK-WT. **(C–D)** Docking of ASP3026 to ALK mutants (C, ALK-D1203N; D, ALK-E1210K) showing a different pose (C atoms in purple). Mutations spots and DFG motif residues are showed in licorice representation (C atoms in cyan and iceblue, respectively).

In order to rationalize the experimental data, with particular reference to sensitivity/resistance to ASP3026, we ran a molecular modelling analysis of N/A mutants. Molecular docking simulations were carried out using GOLD 5.2.2. Before running simulations with ASP3026, the docking protocol was validated by assessing the capability of GOLD to reproduce the crystallographic structure of ceritinib, an analogous ALK inhibitor when compared to ASP3026, in complex with the enzyme (PDB ID: 4MKC) [26]. These preliminary results clearly showed that the software was able to reproduce the crystallographic complex with RMSD value of 0.8 Å (Supplementary Figure S9A). This allowed us to further apply the docking protocol towards the identification of the putative binding mode of ASP3026 within the active site of ALK. The binding of ASP3026 at the ALK active site showed that ASP3026 and ceritinib share a highly similar interaction network (Supplementary Figure S9B). According to the binding mode identified for ASP3026, we hypothesized that the mutations G1128S, E1210K and D1203N could be relevant for the interactions-based recognition process and in the stability of protein/inhibitor complex (Figure 4A). In particular, docking of ASP3026 into E1210K or D1203N ALK mutants showed a preferred solution that was different from that in the WT, suggesting that these mutations may cause an inhibitor shift, increasing the binding energy (Figure 4C–4D). Asp1203 lies at the bottom of the ATP pocket, in close proximity to inhibitors, therefore it is expected to hinder inhibitor binding [27, 28]. Moreover, it is located within the hinge region, adjacent to two residues associated with drug resistance (G1202 and S1206) and it makes a water-mediated hydrogen bond with ADP [29], suggesting that mutations in this position may also affect enzyme kinetics. G1128S lies within the glycine-rich nucleotide-binding region (P-Loop) and seemed to have an effect on the flap of the glycine-rich loop that may result in enhanced ATP binding. The other mutations are too far from the inhibitor-binding site, outside the range of the docking protocol (15 Å) and therefore they could not be used to simulate ASP3026 docking. However, from the structural information available, we hypothesized that they may have an influence on the stability of the hydrophobic pocket of the active site (I1171N/T) as well as on the kinetics of the DFG motif and the activation loop (I1171N/T, F1174I and N1178H) (see Figure 4B). In particular, I1171 seems to be a crucial determinant of enzyme regulation: it is part of the αC helix, which governs activation of protein kinases, and of the hydrophobic spine, another key regulatory element [34]. As postulated in our previous molecular modelling analysis [12], I1171N (and possibly any change at this position) alters the stability of the hydrophobic spine and renders the kinase intrinsically more active by stabilizing the active conformation. We could not devise any explanation for the C1156F mutant data. However, according to recent studies, a change at C1156 may interfere allosterically with the binding of inhibitors by causing conformational changes in the inhibitor binding cavity and a consequent displacement of the compounds [7, 24, 30].

## DISCUSSION

The acquisition of resistance to kinase inhibitors has emerged as a big hurdle in targeted anticancer therapy. The knowledge of resistance profiles associated to each available drug is of great importance in the management of patients. Along these lines, we set out to identify the mechanisms that may lead to ALK inhibitor resistance in ALCL. Point mutations, ALK gene amplification, activation of alternative signaling pathways, as well as yet unknown mechanisms have been recognized thus far [7–11]. In this study, we selected several ALCL cell lines that are able to propagate in the presence of high doses of ASP3026. In all derived cell lines, we identified at least one nonsynonimous ALK kinase domain mutation that rendered the enzyme less susceptible to inhibition, as demonstrated in transfected Ba/F3 cells. SUPM2 resistant cells grew in the presence of 2 μM inhibitor, while it was not possible to reach the same dose with the K299 line. This is reflected by higher RR values displayed by SUPM2-derived mutants. As illustrated by K299R3 and SUPM2R3 cells, it is possible that all resistant cell populations accommodate multiple mutant subclones at low frequency, that may expand under certain circumstances, such as second-line ALKi therapies. It is also likely that moderate N/A overexpression in K299 cells synergizes with point mutations to achieve drug resistance.

In the analysis of BaF-N/A sensitivity to inhibitors, we reported both the RR and TI values for each mutant, along with the raw IC_50_ value. We believe that the two data normalizations are complementary and are both useful: RR index gives a mechanistic/molecular view of the relative sensitivity of a mutant versus the wild-type enzyme; on the other hand, TI values provide a view of the therapeutic impact of a mutation, i.e. how much the mutant becomes similar to non-responding ALK-negative cells: this is related to unspecific toxicity of a drug. In many cases, RR and TI give similar outputs. However, it is possible that even a marked drop in sensitivity, e.g. 10-fold, does not translate in real clinical resistance, due to large therapeutic windows (difference between specific and unspecific activity). This concept is best recapitulated by cross-resistance data with PF-06463922. Although most mutants were attributed a RR > 10, due to very potent activity against wild-type N/A, their sensitivity was still in the low nanomolar range, easily achievable under standard clinical regimens. Therefore, we should be cautious when interpreting these data: although the compound indeed suffers a shift in sensitivity caused by some mutations, physicians may still be able to successfully treat a patient carrying such mutations. Similarly, Friboulet and colleagues reported significant loss of sensitivity to ceritinib for L1196M (in H3122 cells) and C1156Y (BaF3-EML4/ALK) mutants compared to wild-type *in vitro*, yet the two mutations could be targeted *in vivo*, likely due to higher potency of ceritinib compared to crizotinib, at tolerable concentrations [26].

Molecular modelling analysis provided hints to the possible mechanisms leading to the observed resistance to ASP3026. Glycine 1128 is conserved in most kinases. Indeed, the glycine-rich loop in the ATP-binding site is one of the most highly conserved sequence motifs in protein kinases. The same residue was found mutated in neuroblastoma patients (G1128A) [31], where it is thought to contribute to kinase hyper-activation by making the P-Loop more rigid and thus facilitating ATP access to the active site [28]. We observed a Gly to Ser substitution (G1128S): according to Hemmer et al. both Gly-to-Ser and Gly-to-Ala mutations in PKA P-loop greatly increased ATP hydrolysis and phosphate transfer to a peptide substrate [32]. Furthermore, G1128 corresponds to G254 of ABL, which is flanked by two hotspots of imatinib-resistant BCR/ABL mutations [33].

Cysteine 1156 has previously been implicated in resistance to crizotinib, when mutated to Tyr (C1156Y) [7]. Phenylalanine is a tyrosine lacking the hydroxyl group. Therefore, they are structurally similar, but have different polarity. ALK fusion proteins carrying the crizotinib-resistant C1156Y mutation are reported to be sensitive to AP26113 [34], alectinib [19], and (partially) to ceritinib [26]. In our cells, C1156F alone was sufficient to cause resistance to ASP3026, but surprisingly not to crizotinib, while resistance increased significantly when combined *in cis* with D1203N mutation, which has been observed in an *in vitro* resistance screening with crizotinib and NVP-TAE684 [27]. Interestingly, C1156F also affected inhibition by the other compounds, thus showing a very different behavior compared to C1156Y [19, 23, 34]. How the simultaneous presence of C1156F and D1203N mutations acts synergistically to induce resistance is at present unclear. The clone harboring a single C1156F substitution has likely arisen earlier (probably at lower ASP3026 concentration) and has subsequently acquired the second hit at D1203, since we observed rare C1156F single mutants but never found D1203N alone, in our clones. Indeed, the double C1156F/D1203N mutant shows higher RR to ASP3026 compared to C1156F.

Ile1171 has been identified as a mutational hotspot in various models of resistance to ALK inhibitors. We previously described an I1171N change in crizotinib-resistant cells *in vitro* [12] and in one relapsed ALCL patient [35]. This mutant proved resistant to NVP-TAE628, too. Recently, I1171T/N/S mutations developed in three alectinib-resistant NSCLC patients [36, 37]. The I1171T mutant was identified in crizotinib-resistant cells [38] and xenografts [26] that showed a shift in sensitivity to both crizotinib (4-fold) and ceritinib (3-fold) in Ba/F3 cells, compared to wild-type. However, regression of tumors harboring such mutation were obtained *in vivo* by ceritinib. Recently, the I1171N mutant was described as sensitive to ASP3026 in a 293T cell model [17]. However, the authors could only obtain a partial decrease in NPM/ALK phosphorylation at a high drug concentration. Finally, both I1171T and I1171N mutants were described in an *in vitro* mutagenesis screen with crizotinib [39]. We found three different substitutions at I1171 among ASP3026-resistant cells, again indicating that I1171 is a critical residue controlling sensitivity to ALK inhibitors. The three changes are not equivalent: threonine makes the residue smaller, thus creating more space; on the other hand, serine and asparagine are polar residues. According to our analysis, I1171 mutations affect both the inhibitor binding site and the enzyme kinetics. This is confirmed by the finding that I1171N is both an activating mutation in neuroblastoma patients [31] and a drug-resistant mutant in ALCL and NSCLC patients [35–37].

Phenylalanine 1174 lies at the C-terminal end of the αC helix. According to Bossi et al., it is the core residue of a hydrophobic network that controls kinase activation [28]. Mutations at this position would facilitate the formation of transient structures that promote the active conformation of the kinase. Indeed, F1174L is a frequent mutation in neuroblastoma [31, 40]. In addition, F1174L was identified in a crizotinib-resistant IMT patient with RANBP2-ALK translocation [9]. Biochemical analysis showed that F1174L has a significantly higher catalytic efficiency and higher affinity for ATP compared to wild-type enzyme, which may explain why it is both an activating and a resistance mutation [29, 41]. We observed a F1174I substitution, which conferred mild resistance to ASP3026 but not to crizotinib, nor to other compounds. In fact, it only represented a minor population within SUPM2R3 cells, that might have been selected at lower doses and then overcome by other, more resistant mutants at the final ASP3026 concentration. To make a direct comparison between Leu and Ile substitutions, we established a BaF-N/A-F1174L cell line and found that it provides limited resistance to crizotinib (RR = 3), suggesting that F1174 mutants in general may be easily overcome by moderate drug dose increase (Supplementary Figure S10). Furthermore, a F1174C change has been observed in K299 cells resistant to alectinib [38], although the mutation was not further validated by ectopic expression in Ba/F3 cells. Yet another substitution, F1174V, was recently isolated from a NSCLC patient who progressed on crizotinib [42] and from our AP26113-resistant cells [13]. Early clinical data with ceritinib showed progression of drug-resistant NSCLC disease via acquisition of F1174V or F1174C mutations [26].

In our second-line therapy simulation, the novel inhibitor PF-06463922 [22] showed superior therapeutic value, due to a very large therapeutic window, which allowed the use of a more efficacious concentration. Inhibitor concentrations used for this analysis were adjusted according to their effects on Ba/F3 cells: they cause no toxicity to IL3-driven parental cells, while inhibiting > 99% BaF-N/A-WT cells, and are close or above the calculated IC_50_ for BaF-N/A-C1156F/D1203N and BaF-N/A-E1210K mutants, which represented the most frequent clones within SUPM2R3 population. Indeed, SUPM2R3 cell pool evolved according to the sensitivities predicted by BaF-N/A transfectants, except for PF-06463922, which did not allow the outgrowth of any clone. The sensitivity of E1210K and C1156F/D1203N mutants to ceritinib or AP26113 is not very different (Tables 3–4), yet the SUPM2R3 polyclonal culture evolved in opposite directions under the two drugs. Either this is a random effect of genetic drift, or the small differences in RR observed are ultimately sufficient to drive evolution in one direction or the other. Furthermore, it should be noted that this second-line selection was run under different conditions compared to the initial ASP3026-driven selection, in that we used relatively higher drug concentrations in a shorter period, allowing for fast clonal evolution. For crizotinib, the used dose corresponds to mean trough plasma concentration measured in patients [43, 44], while for ceritinib and alectinib these values are reported to be higher [20, 45], which may suggest that we underestimated their therapeutic potential (Supplementary Table 3). However, it is difficult to compare *in vitro* and *in vivo* effects of drugs. For example, steady state plasma concentration of alectinib at the recommended dose of 300 mg is about 1 μM [45], but this was clearly toxic to Ba/F3 cells in our model, therefore it could not be taken as surrogate of a ‘safe’ treatment.

In conclusion, this work confirms that resistance to ALK inhibition is a relatively common event. However, the more compounds are available, the higher the probability that at least one drug will be able to overcome resistant disease. Interestingly, many of the mutated residues identified here that conferred resistance to ASP3026 (Cys1156, Ile1171, Phe1174, Asp1203, Glu1210) have previously been linked to resistance to other inhibitors, thus suggesting that the spectrum of possible drug-refractory mutations in ALK kinase is likely limited.

## METHODS

### Cell lines and compounds

Karpas-299, SUP-M2 and Ba/F3 cell lines were purchased from DSMZ, where they are routinely verified using genotypic and phenotypic testing to confirm their identity. The cells were maintained in RPMI-1640 plus 10% fetal bovine serum and antibiotics, in a humidified chamber at 37°C, with 5% CO_2_. In addition, the medium for Ba/F3 parental cells (transfected with empty pcDNA3 vector) was further supplemented with CHO cells supernatant as a source of IL-3. Human drug-resistant cell lines were maintained in the presence of the corresponding compound at the highest concentration under which they were selected. Fresh medium supplemented with drug was provided every three days.

ASP3026 was kindly provided by Astellas Pharma Inc.; crizotinib (PF-02341066) and PF-06463922 [22, 46] were obtained from Pfizer Inc.; ceritinib (LDK378) [20] was from Novartis AG; AP26113 [21, 34] was provided by ARIAD Pharm.; alectinib (CH5424802) [19] was purchased from Selleck Chem. All compounds were dissolved in DMSO at 10 mM, aliquoted and stored at −20°C until use.

### Site-directed mutagenesis and generation of transfected cell lines

The pcDNA3.0 vector containing wild-type NPM/ALK (pcDNA3-NA) was kindly provided by Dr. S. W. Morris (St Jude Research Hospital, Memphis, TN). Mutagenesis was run as described [12], using oligonucleotides reported in Supplementary Table 4. Ba/F3 cells were stably transfected with wild-type or mutated pcDNA3-NA as previously described [12].

### PCR and DNA sequencing

Total RNA was extracted from 10^7^ cells using TRIzol^®^ reagent, following manufacturer's instructions, and reverse transcribed using MultiScribe™ Reverse Transcriptase (Life Technologies) and random hexamers. Quantitative real-time PCR analysis was run as described [12]. For mutation analyses, the NPM-ALK kinase domain region was amplified with High Fidelity Taq Polymerase (Roche) using the following primers: NPM1-forward 5′-TGCATATTAGTGGACAGCAC-3′ and ALK-reverse 5′-GACTCGAACAGAGATCTCTG-3′. Amplicons were purified from agarose gel and either directly sequenced by Sanger method at Eurofins Genomics (Germany) or cloned using the TOPO TA Cloning System (Invitrogen). TOPO-cloned fragments were individually sequenced by Sanger technique. ALK sequence numbering refers to GenBank ID NM_004304.

### Western blotting

The cells were typically treated for 4 hours with vehicle or inhibitors and harvested. After wash with PBS, cell pellets were lysed and loaded on SDS-PAGE as described [12]. The following primary antibodies were used: anti-phosphorylated (p-)ALK (Y1604), total ALK (clone 31F12) and p-STAT3 (Y705) were from Cell Signaling Technologies; anti-β-actin was purchased from Sigma-Aldrich; anti-STAT3 from Millipore; a second anti-ALK antibody (ALK-1) [47] was kindly provided by Prof. Karen Pulford (University of Oxford). Secondary anti-mouse and anti-rabbit antibodies were obtained from Bio-Rad and diluted 1:3000.

### Proliferation assay

The cells were seeded in round-bottom 96-well plates (10,000/well) in the presence of serial dilutions of compounds in DMSO (0.5% final DMSO concentration) and incubated for 72 hours. During the last 8 hours of incubation, the cells were pulsed with [Methyl-^3^H]-Thymidine and then harvested onto glass fiber filtermats using a Tomtec Cell Harvester. Filters were counted with a Wallac Microbeta 1405 Scintillation Beta Counter. Radioactivity associated to each sample is proportional to the amount of labelled thymidine incorporated into newly synthesized DNA, giving a direct measure of the cell proliferation rate. All values are normalized to vehicle-treated control which is set as 100% proliferation. The Inhibitory Concentration 50 (IC_50_) value is defined as the inhibitor concentration that yields 50% proliferation, relative to control. IC_90_ represents the concentration that causes 90% inhibition.

### Soft-agar colony assay

One thousand cells were seeded in 6-well plates, embedded in 0.3% low-melting agarose, with or without inhibitor, on a 0.5% bottom agar layer, as previously described [48].

### Relative resistance, therapeutic index and statistical analyses

The Relative Resistance (RR) index is defined as the IC_50_ fold increase compared to the value obtained with wild-type cell lines. Therapeutic Index (TI) is the ratio between IC_50_ of Ba/F3 parental cells and IC_50_ of BaF-NPM/ALK transfectants. All dose-response curves and IC_50_ calculations were made by GraphPad Prism software. Sequence alignment was performed using Vector NTI 10.3. Chemiluminescence Western blotting images were acquired and analyzed by Carestream Molecular Imaging software. Relative NPM/ALK expression was calculated by densitometry analysis of anti-ALK signal, normalized on anti-actin bands. All data shown in the article are representative of at least three independent experiments.

### Molecular modelling

The model of ALK was constructed by removing all water molecules and ligands from the X-ray structure (PDBcode: 4MKC) and by adding the hydrogen atoms using SYBYL-X 2.1.1 (Tripos Associates Inc, USA) [49]. The 3D models of ligands were built by using SYBYL-X 2.1.1. Docking simulations were carried out by means of GOLD, version 5.2.2 (Cambridge Crystallographic Data Centre, http://www.ccdc.cam.ac.uk/Solutions/GoldSuite/pages/GoldSuite.aspx). GOLD adopts a search genetic algorithm to generate lowest binding ligand-protein complex energies. Genetic algorithm default parameters were set: the population size was 100, the selection pressure was 1.1, the number of operations was 105, the number of islands was 5, the niche size was 2, migrate was 10, mutate was 95, and crossover was 95. Docking calculations were computed to obtain 100 randomly seeded runs for each ligand. Binding-site cavity was set as a spherical region of 15 Å radius centered on the phenolic N atom of backbone of the M199 residue. To evaluate the single poses resulted by search algorithm GoldScore scoring function was used.

## SUPPLEMENTARY FIGURES AND TABLES


