# Supplementary material for: NPM/ALK mutants resistant to ASP3026 display variable sensitivity to alternative ALK inhibitors but succumb to the novel compound PF-06463922
Source: Oncotarget. 2015 Jan 30;6(8):5720–34. doi: 10.18632/oncotarget.3122 (PMC4467397; doi:10.18632/oncotarget.3122)
Supplement: Supplementary file 1 [file oncotarget-06-5720-s001.pdf]

SUPPLEMENTARY FIGURES AND TABLES

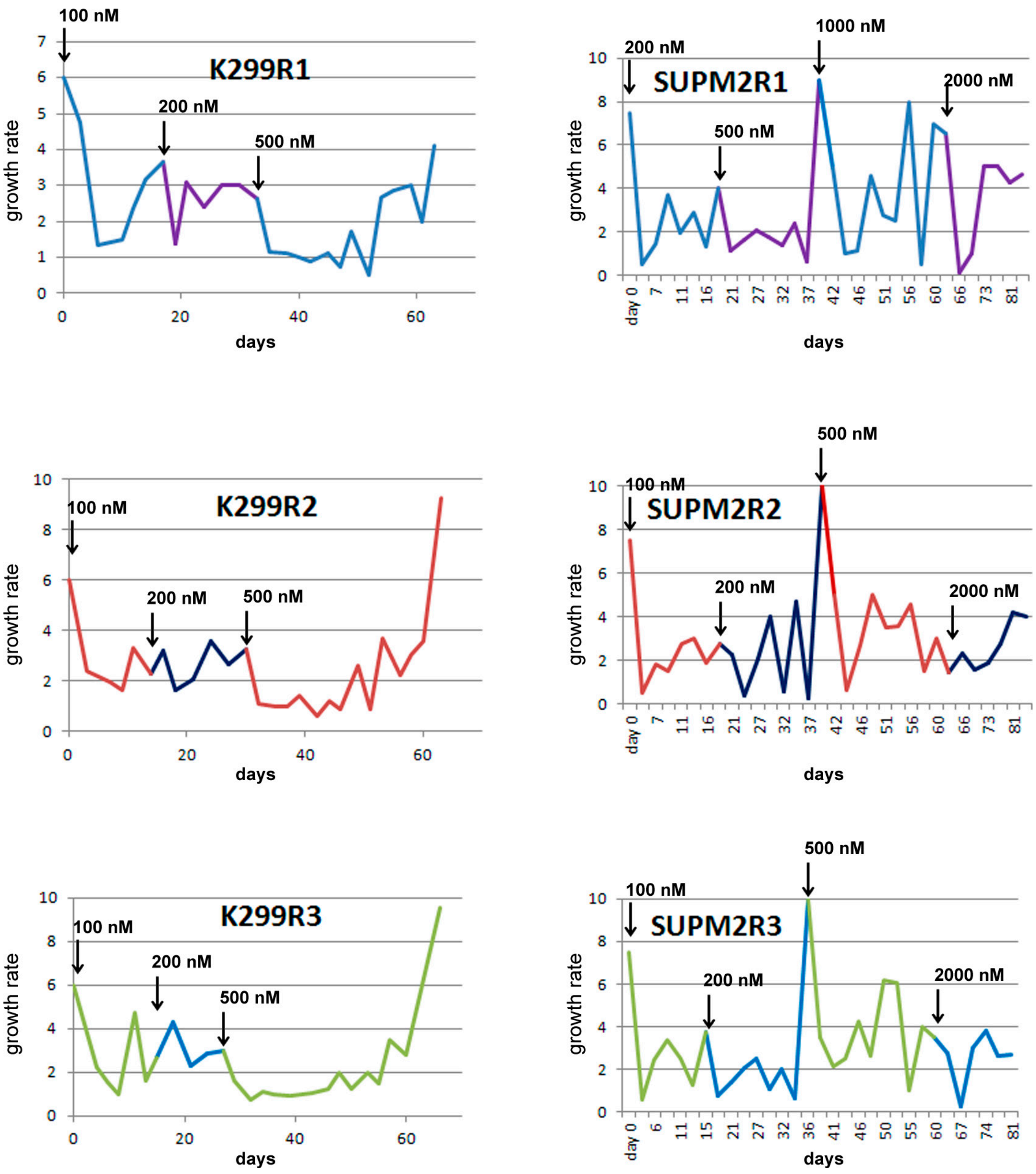

Supplementary Figure S1: Growth rates of cell cultures under drug selection. Growth rate was calculated as the variation in cellular concentration during each time interval. Arrows indicate the time of shift in drug concentration. The curves change color at each drug increase, to facilitate reading.

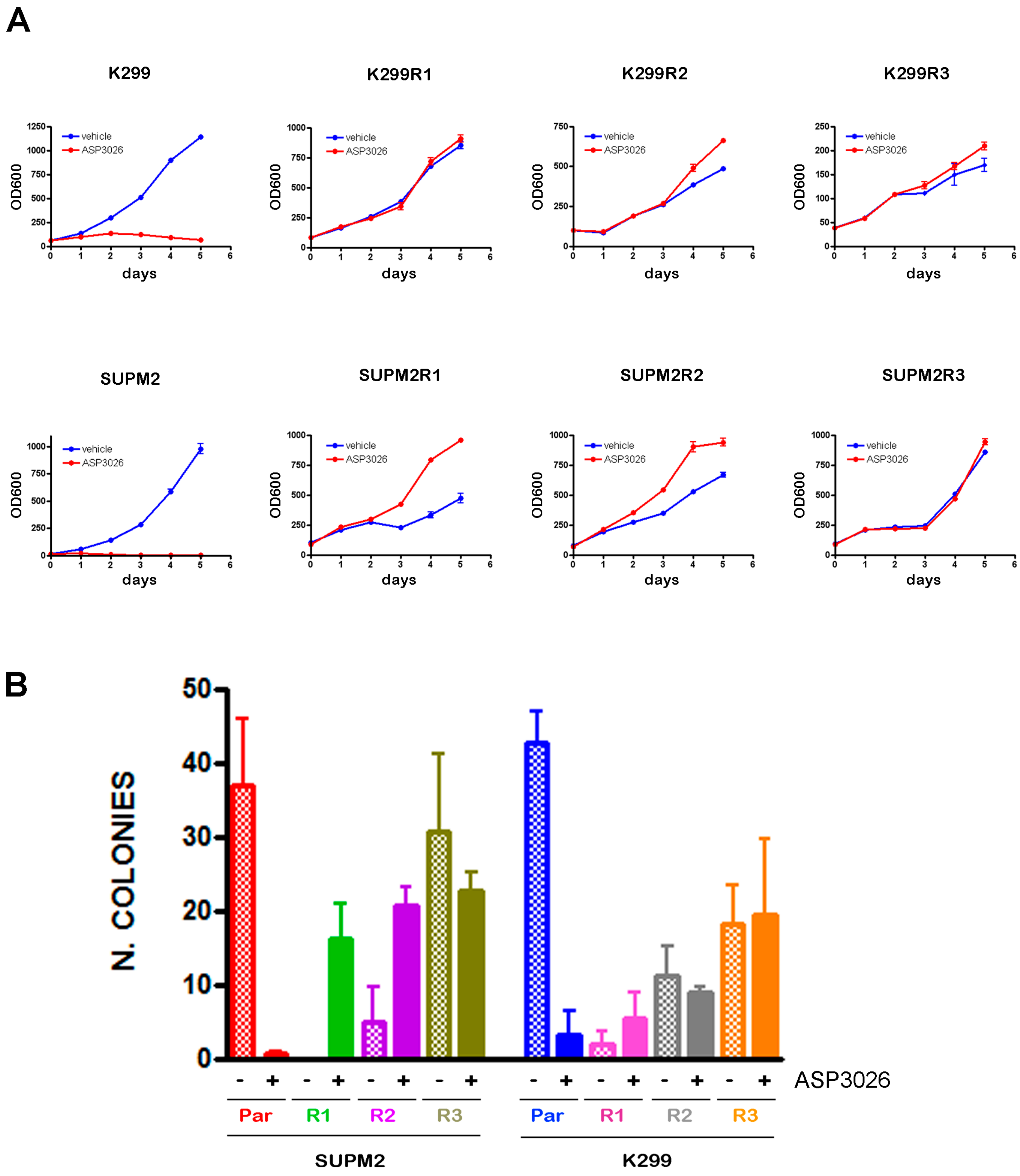

Supplementary Figure S2: Growth properties of parental and ASP3026-resistant SUPM2 and K299 cells. **(A)** In vitro growth kinetics in liquid culture. A time-course MTS viability assay was run in the absence (blue lines) or in the presence (red lines) of ASP3026. **(B)** Anchorage-independent growth was investigated by soft-agar colony assay with (solid bars) or without (cross-hatched bars) ASP3026. The data represent two independent experiments.

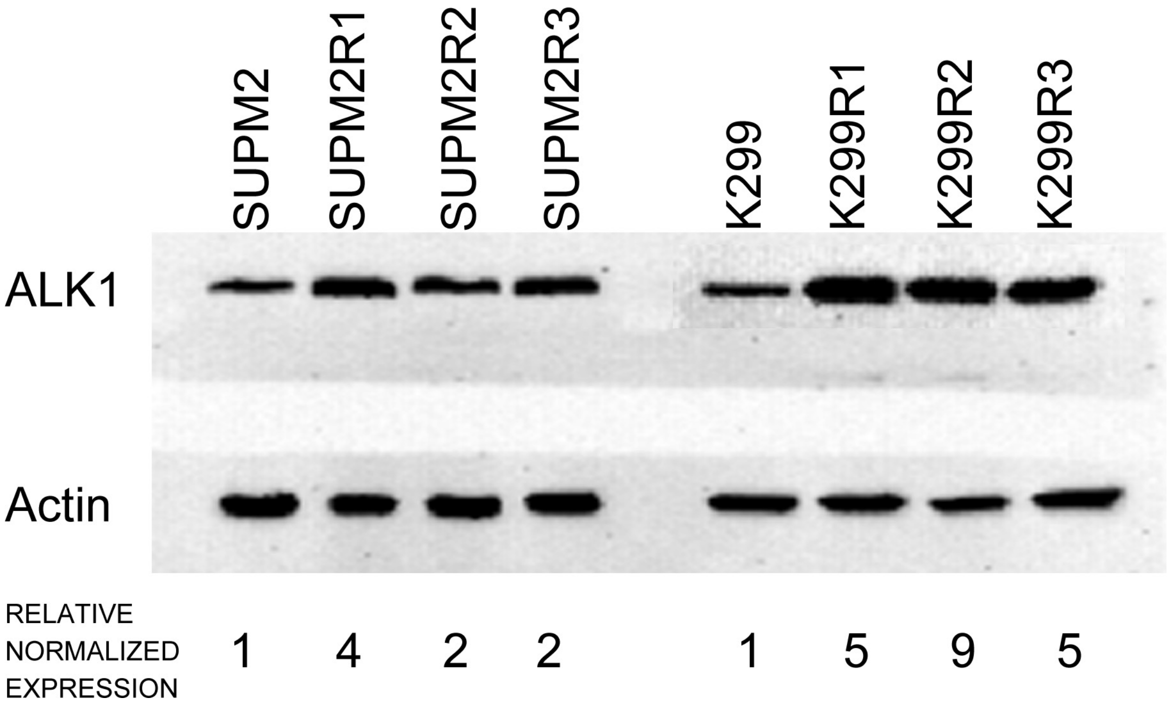

Supplementary Figure S3: Western blot analysis of ALK expression in ASP3026-resistant cells. Total cell lysates were probed with ALK1 antibody (ref. 47). Actin is shown as a loading control. Densitometry analysis of relative band intensity is shown below the blot.

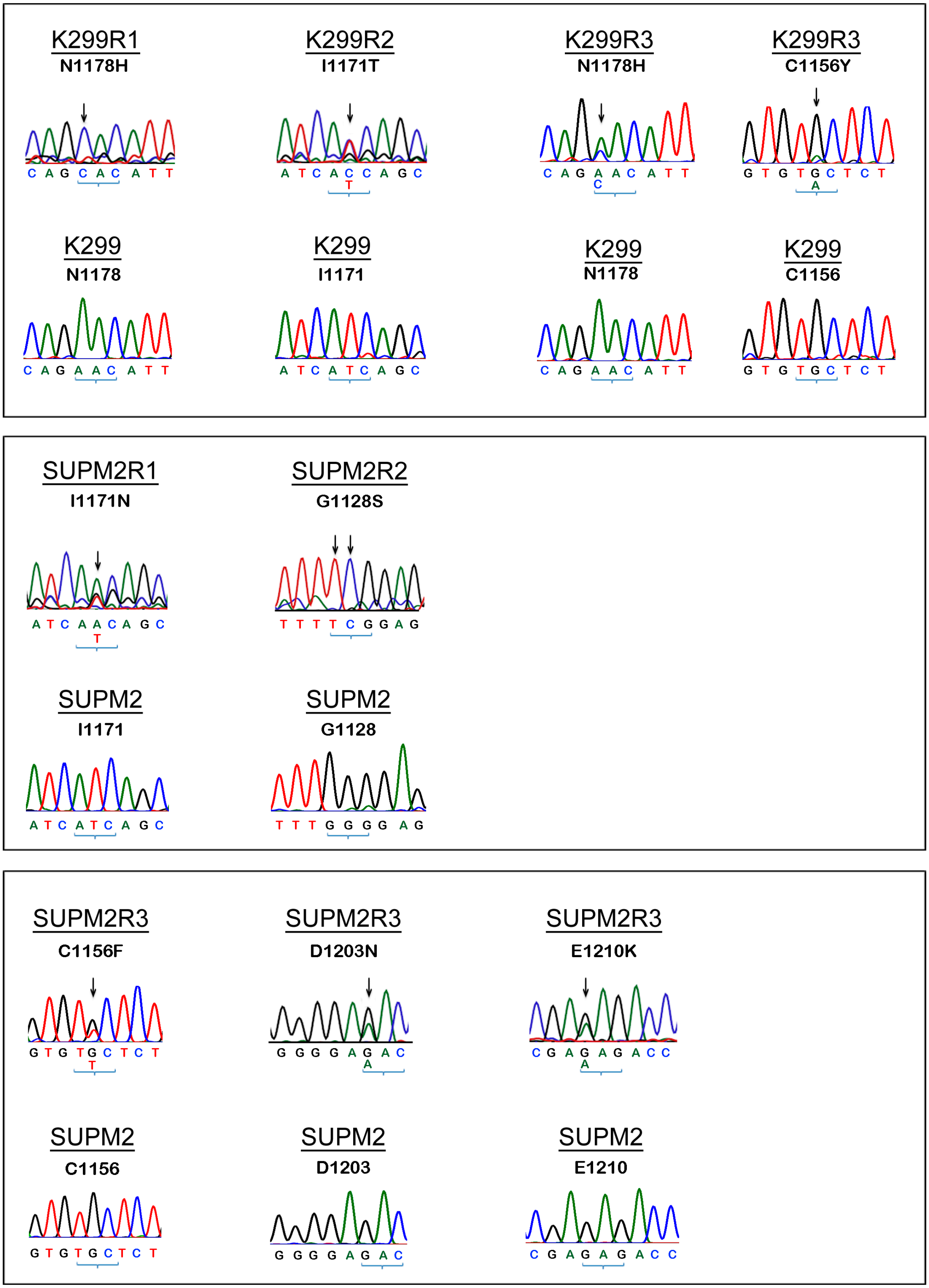

Supplementary Figure S4: Chromatograms representative of mutations found in ASP3026-resistant cells. The corresponding sequence from parental cells is shown below each mutant, for a comparison.

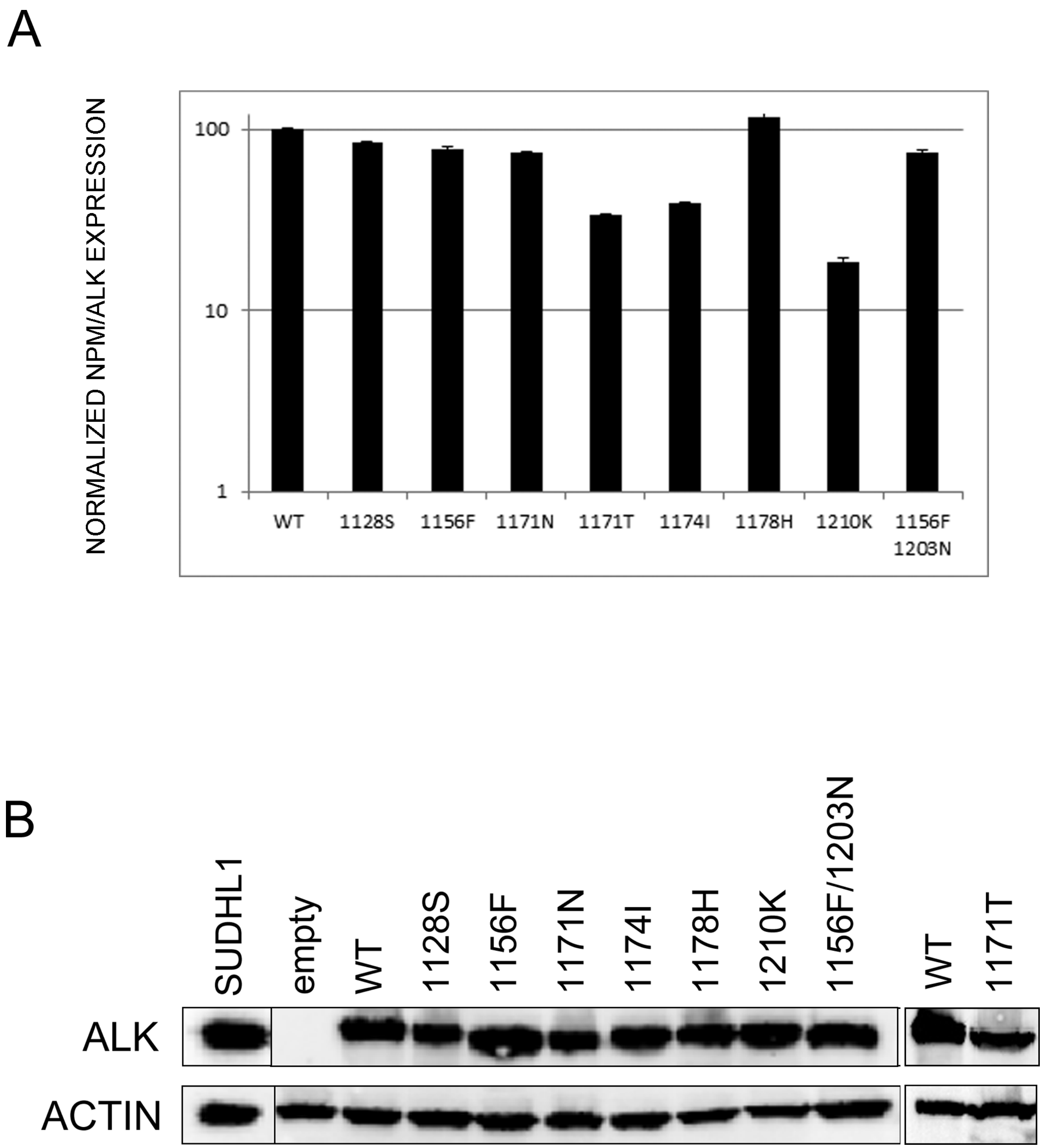

Supplementary Figure S5: Ectopic expression of NPM/ALK mutants in transfected Ba/F3 cells. at RNA **(A)** and protein **(B)** level. SUDHL1 lysate was run in parallel for a comparison. A line is drawn where the gel was cut. The 1171T mutant was run on a separate gal alongside WT.

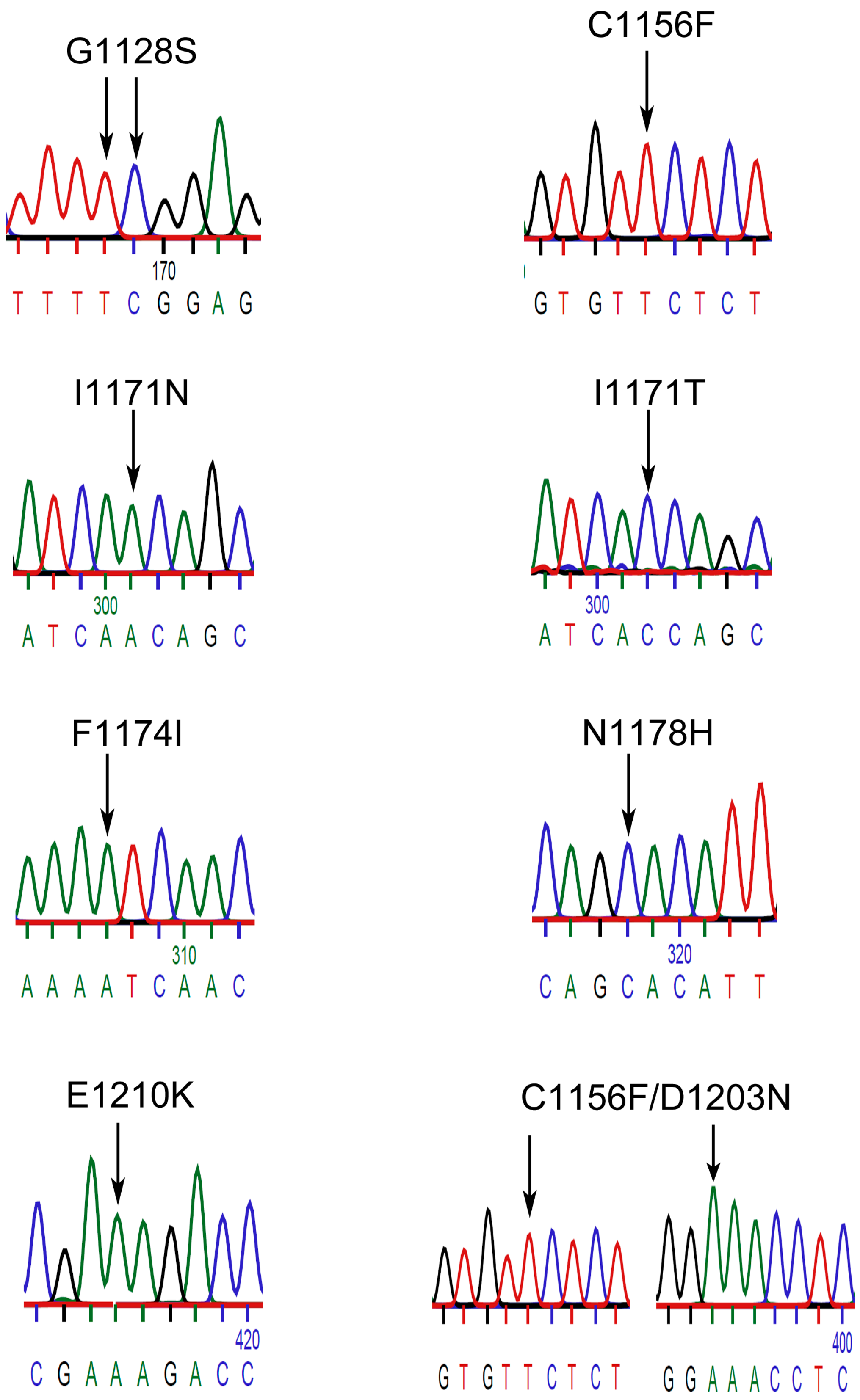

Supplementary Figure S6: Sequencing of mutant N/A expressed in the various Ba/F3 transfected cell lines.

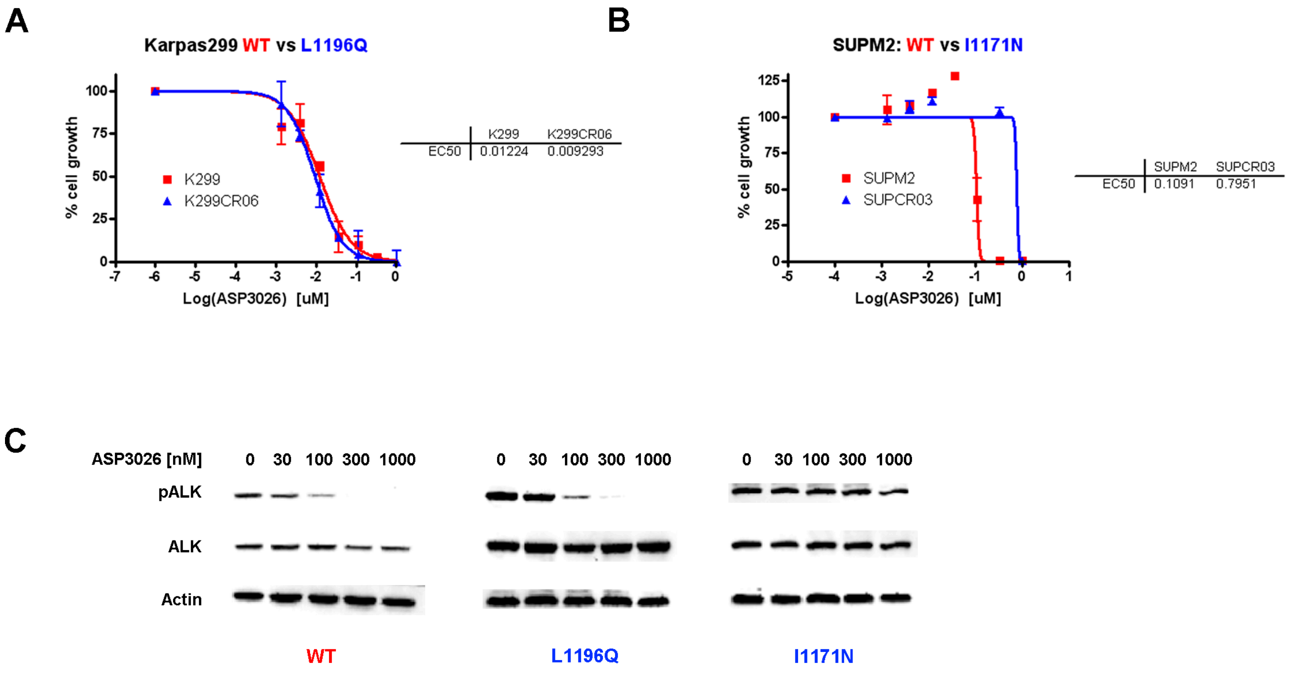

Supplementary Figure S7: Analysis of cross-resistance to ASP3026 of previously described crizotinib-resistant cell lines (ref. 12). Cells carrying the L1196Q substitution are fully sensitive to ASP3026, while they showed 40 × RR to crizotinib in the original report, supporting non-overlapping resistance profiles. Cells with the 1171N mutation, by contrast, show a similar degree of resistance to both drugs (7-fold) in line with this mutant being selected in both cases.

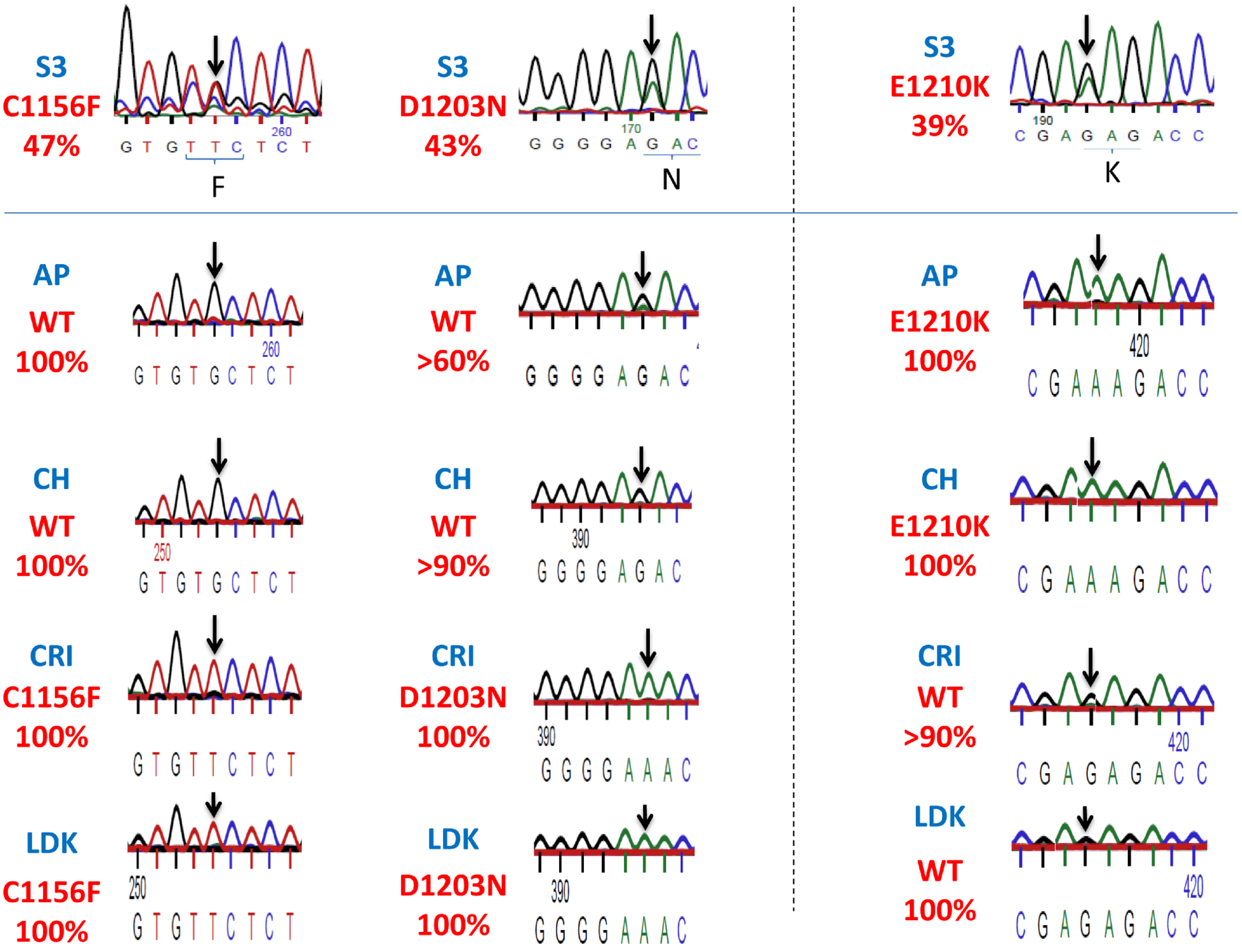

Supplementary Figure S8: Sequencing of second-line treatment-resistant cells. SUPM2R3 sequence (pre-treatment) is reported at the top. The sequence of the corresponding regions after second-line treatments is shown below the line. Arrows point at the mutated nucleotides.

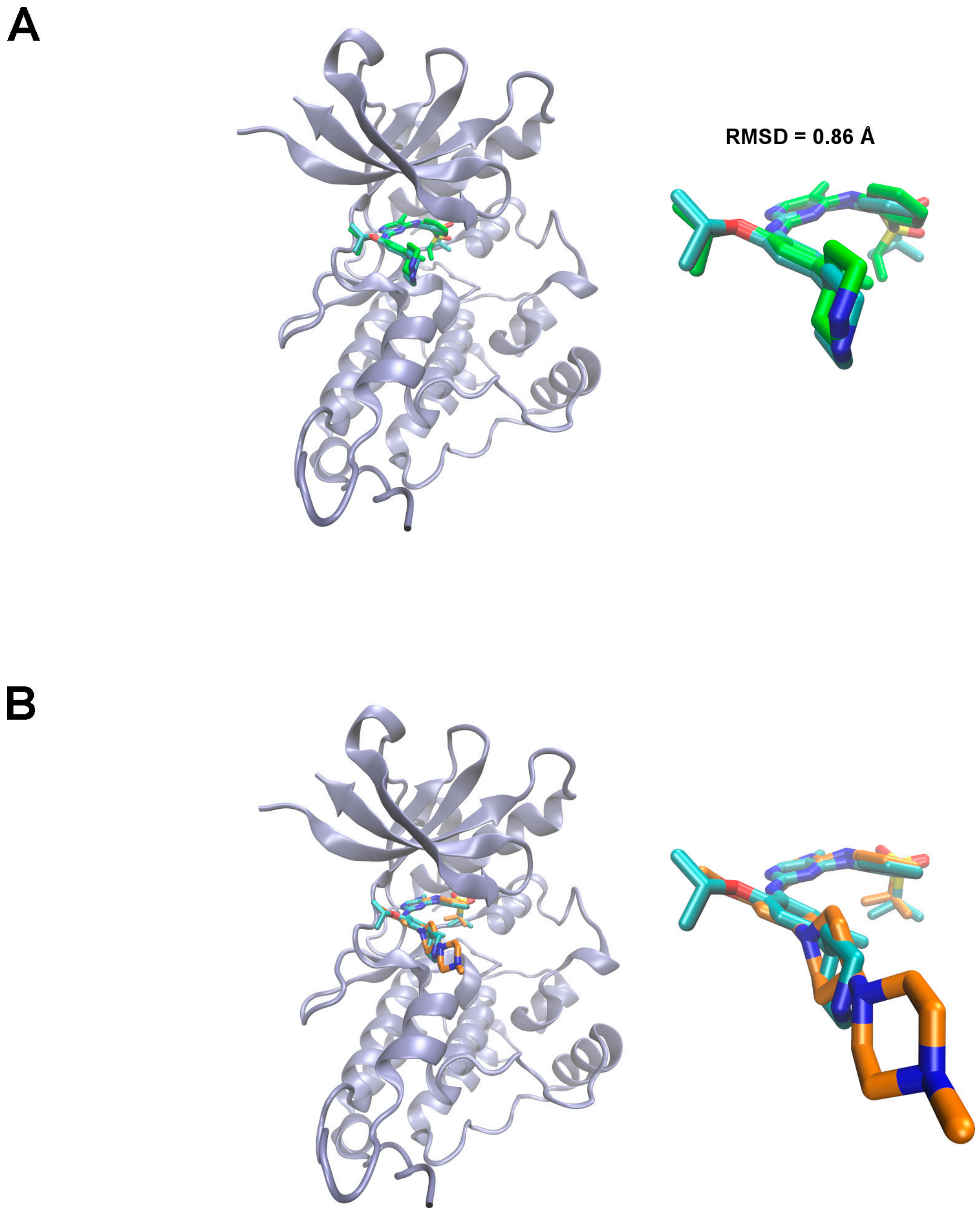

Supplementary Figure S9: Validation of the docking protocol. **(A)** Overlap of the crystallographic pose of ceritinib (C atoms in cyan) and its docking pose (C atoms in green) within the active site of ALK (PDB code: 4MKC). **(B)** Overlap of the crystallographic pose of ceritinib (C atoms in cyan) and the low energy docking pose of ASP3026 (C atoms in orange) within the active site of ALK (PDB code: 4MKC).

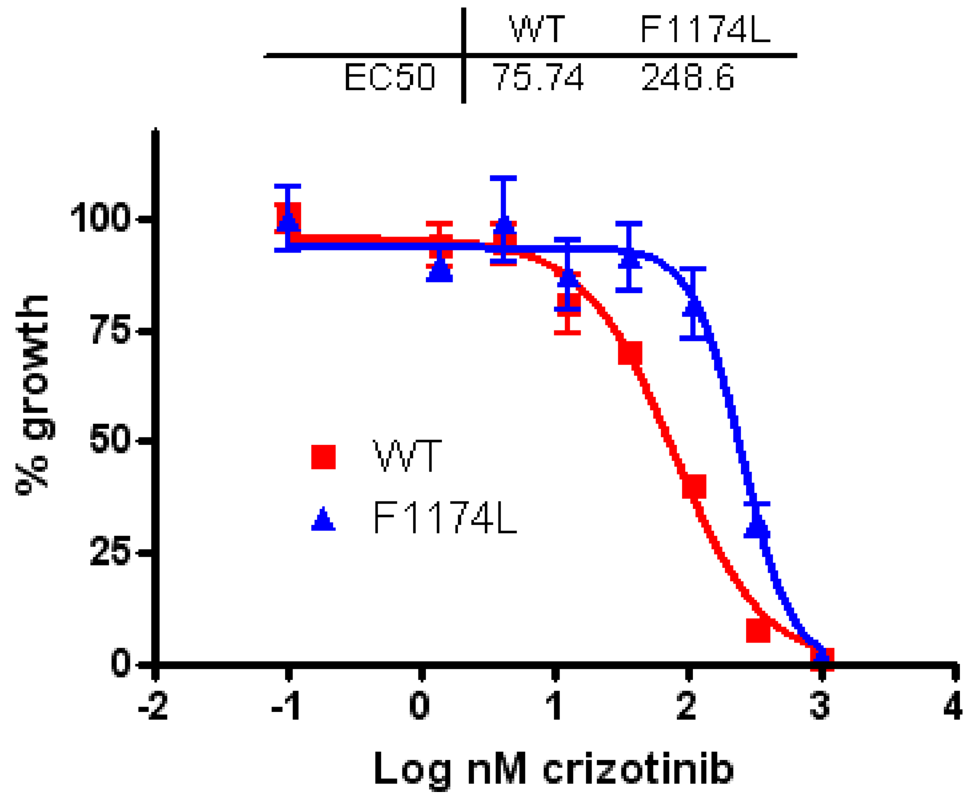

Supplementary Figure S10: Dose-response curves obtained by 3H-Thymidine incorporation assay with Ba/F3-NPM/ALK wild-type and F1174L treated with crizotinib. IC50 values are reported at the top of the graph.

Supplementary Table S1: NPM/ALK mutations detected at frequency >1% in the K299R3 cell line, by ultradeep sequencing of the ALK kinase domain.

Nucleotide change	Aminoacid change	Mutated clones/total reads	Frequency (%)
AAC -> CAC	N1178H	3904/7890	49.48
TGC -> TAC	C1156Y	1755/7641	22.97
CTG -> ATG	L1196M	545/7988	6.82
ACG -> AAG	T1151K	189/7931	2.38
CCG -> CAG	P1215Q	179/7924	2.26
ATC -> AGC	I1105S	73/5359	1.36
GAC -> GGC	D1225G	102/7961	1.28
GAC -> GGC	D1232G	84/7876	1.07
CGC -> CAC	R1214H	76/7244	1.05
GCT -> CCT	A1230P	82/7857	1.04
CTG -> CGG	L1108R	82/7997	1.03
AAC -> ATC	N1394I	79/7825	1.01

Supplementary Table S2: NPM/ALK mutations detected at frequency >1% in the SUPM2R3 cell line, by ultradeep sequencing of the ALK kinase domain.

Nucleotide change	Aminoacid change	Mutated clones/total reads	Frequency (%)
TGC -> TTC	C1156F	3563/7338	48.56
GAG -> AAG	E1210K	3110/7993	38.91
GAC -> AAC	D1203N	2830/7664	36.93
TTC -> ATC	F1174I	784/7976	9.83
ATC -> AGC	I1171S	389/7985	4.87
ATT -> ATG	I1119M	256/7992	3.20
TTC -> TGC	F1174C	184/7977	2.31
ACG -> ATG	T1151M	173/7792	2.22
TCC -> CCC	S1219P	112/7986	1.40
GAG -> GGG	E1129G	56/5428	1.03
TTC -> CTC	F1193L	80/7988	1.00

Note: deep-seq does not discriminate single from compound mutations.

Supplementary Table S3: ALK inhibitors plasma concentrations reported in literature.

DRUG	Dose	Plasma concentration [nM]	References
**Crizotinib**	250 mg/kg BID	530-649¶	43, 44
**Alectinib**	300 mg/kg BID	880-959¶	45
**Ceritinib**	750 mg/kg QD	1400*	20
**AP26113**	180 mg/kg QD	1000-2000¶	34
**PF-06463922**	0.3-10 mg/kg BID	250-2500†	46

¶trough plasma levels (C_{trough}) in patients.
*peak plasma levels (C_{max}) in patients.
†peak plasma levels (C_{max}) in mice.

Supplementary Table S4: Oligonucleotides for site-directed mutagenesis. The mutated nucleotides are underlined.

Mutation	Sense (5′-3′)	Antisense (5′-3′)
**G1128S**	GCCATGGCGCCTTTTCGGAGGTGTATGAAG	CTTCATACACCTCCGAAAAGGCGCCATGGC
**C1156F**	GACGCTGCCTGAAGTGTTCTCTGAACAGGACGAAC	GTTCGTCCTGTTCAGAGAACACTTCAGGCAGCGTC
**I1171N**	CATGGAAGCCCTGATCAACAGCAAATTCAACCACC	GGTGGTTGAATTTGCTGTTGATCAGGGCTTCCATG
**I1171T**	CATGGAAGCCCTGATCACCAGCAAATTCAACCACC	GGTGGTTGAATTTGCTGGTGATCAGGGCTTCCATG
**F1174I**	GCCCTGATCATCAGCAAAATCAACCACCAGAACATTG	CAATGTTCTGGTGGTTGATTTTGCTGATGATCAGGGC
**N1178H**	GCAAATTCAACCACCAGCACATTGTTCGCTGCATTG	CAATGCAGCGAACAATGTGCTGGTGGTTGAATTTGC
**D1203N**	CTCATGGCGGGGGGAAACCTCAAGTCCTTCC	GGAAGGACTTGAGGTTTCCCCCCGCCATGAG
**E1210K**	CAAGTCCTTCCTCCGAAAGACCCGCCCTCGCCC	GGGCGAGGGCGGGTCTTTCGGAGGAAGGACTTG
